# Sugar intake from sweet food and beverages, common mental disorder and depression: prospective findings from the Whitehall II study

**DOI:** 10.1038/s41598-017-05649-7

**Published:** 2017-07-27

**Authors:** Anika Knüppel, Martin J. Shipley, Clare H. Llewellyn, Eric J. Brunner

**Affiliations:** Department of Epidemiology and Public Health, University College London, London, WC1E 6BT UK

## Abstract

Intake of sweet food, beverages and added sugars has been linked with depressive symptoms in several populations. Aim of this study was to investigate systematically cross-sectional and prospective associations between sweet food/beverage intake, common mental disorder (CMD) and depression and to examine the role of reverse causation (influence of mood on intake) as potential explanation for the observed linkage. We analysed repeated measures (23,245 person-observations) from the Whitehall II study using random effects regression. Diet was assessed using food frequency questionnaires, mood using validated questionnaires. Cross-sectional analyses showed positive associations. In prospective analyses, men in the highest tertile of sugar intake from sweet food/beverages had a 23% increased odds of incident CMD after 5 years (95% CI: 1.02, 1.48) independent of health behaviours, socio-demographic and diet-related factors, adiposity and other diseases. The odds of recurrent depression were increased in the highest tertile for both sexes, but not statistically significant when diet-related factors were included in the model (OR 1.47; 95% CI: 0.98, 2.22). Neither CMD nor depression predicted intake changes. Our research confirms an adverse effect of sugar intake from sweet food/beverage on long-term psychological health and suggests that lower intake of sugar may be associated with better psychological health.

## Introduction

Sugar consumption is increasingly discussed as an intervention target to reduce prevalence of obesity, diabetes and other non-communicable diseases^1, 2^. In Britain, adults consume approximately double, and in the U.S. triple, the recommended level of added sugar for additional health benefits (5% of energy intake), with sweet foods and drinks contributing three-quarters of the intake^1, 3, 4^. Meanwhile, major depression is predicted to become the leading cause of disability in high income countries by 2030^5^.

Higher sugar consumption was linked to higher depression prevalence in several ecological and cross-sectional studies^6–8^. To date, few studies have investigated the prospective association of sweet food and beverage intake with depression^9–12^. Although all studies found an increased risk of depression with higher baseline consumption of added sugars, soft drinks, juices and pastries; none examined the role of ‘reverse causation’ in producing the observed association. Reverse causation refers, in this context, to the possibility that a mood disorder may lead to higher sugar intake, so that the diet-mental health association is wholly or partly the result of poor mental health rather than of high sugar intake^13–15^. A prospective study with repeat measures of food intake and mental health provides the opportunity to examine the bidirectional nature of the association, and to contribute novel evidence on the effect of sugar dense diet on depression in the general population.

There are several plausible biological explanations for an association of habitual sugar intake and subsequent risk of depression, in the long-term. Firstly, low levels of the growth factor brain derived neurotrophic factor (BDNF) have been discussed as facilitating neurogenesis and hippocampal atrophy in depression^16^. Rodents fed high-fat high-sugar diets, but not high-fat diets only, show a decrease in BDNF level^17–19^, which could be a mechanistic link between diets high in sugar and depression. Secondly, carbohydrate consumption has been associated with increased circulating inflammatory markers, which may depress mood^20, 21^. Thirdly, high sugar diets could induce hypoglycaemia through an exaggerated insulin response and thereby influence hormone levels and potentially mood states^22^. Fourthly, addiction-like effects of sugar suggest dopaminergic neurotransmission mechanisms might connect frequent sugar intake with depression^23–25^. Lastly, obesity could be a mediating factor between a sugar-dense diet and depression^26, 27^ not only via inflammatory but also psychosocial factors like weight discrimination^28^.

The aim of this study is to investigate whether sugar intake from sweet food/beverages is positively associated with the risk of both incident and recurrent mood disorders, and to establish the role of the reverse effect in the Whitehall II cohort, using prospective, repeat measures data collected over a 22 year period.

## Methods

### Study cohort

The Whitehall Study II consists of non-industrial civil servants, who were recruited in London at age 35 to 55 years during 1985–1988 (phase 1). The initial sample size was 10,308 individuals (33.1% female and 66.9% male). The participants were followed up via questionnaire in 1989–1990 (phase 2), 1991–1993 (phase 3), 1995–1996 (phase 4), 1997–1999 (phase 5), 2001 (phase 6), 2003–2004 (phase 7), 2006 (phase 8), 2008–2009 (phase 9) and 2012–2013 (phase 11). In phases 1, 3, 5, 7, 9 and 11 they were additionally invited for screening in a research clinic^29^. Phase 10 (2011) consisted of a smaller sample of participants used for a pilot study. The study was approved by the Joint UCL/UCLH Committee on the Ethics of Human Research and carried out in accordance with the ethical principles set out in the Declaration of Helsinki. Further all participants have been asked for informed consent at every follow-up.

### Ascertainment of sugar intake from sweet food/beverages

Diet was assessed at phases 3, 5, 7 and 9 using a 127-item machine-readable semi-quantitative food frequency questionnaire (FFQ) which originates from the tool used in the US Nurses’ Health Study, a self-administered questionnaire on habitual diet over the past 12 months^30, 31^. In order to reflect most diets in the UK it has been modified and anglicized^32^. This FFQ has been validated against a 7 day diet diary in a stratified random sample of 865 participants in the Whitehall Study II at collection phase 3^30^. Sweet food and beverage intake was measured with 15 items such as cakes, biscuits, added sugar to coffee or tea, and fizzy soft drinks (see Supplementary Table S1). Sugar intake was calculated by multiplying sweet food/beverage consumption frequencies per day by their sugar content and portion size based on *McCance and Widdowson’s The Composition of Foods, 5th edition* ^33^.

### Depressive symptom assessment

The 30-item General Health Questionnaire (GHQ) measures depressive and somatic symptoms over the past two weeks^34^. Caseness was defined as reporting ≥5 symptoms and is referred to as common mental disorder (CMD). This measure was included in follow-up questionnaires at all phases apart from phase 4. In addition, the 20-item Center of Epidemiologic Studies Depression Scale (CES-D), a self-report measure of depressive symptoms in the general population over the past week^35^, was administered at phases 7, 9 and 11. Individuals scoring ≥16 were considered cases of depression^36^. Lastly, a clinical interview using the Revised Clinical Interview Schedule (CIS-R) was administered at phase 11 with participants assessed according to International Classification of Diseases (ICD-10) F32 criteria. The computerized self-completion version of the CIS-R included questions on depressive symptoms that were present for at least 2 weeks^37–39^. The GHQ and CES-D have been validated against the CIS-R in this cohort and showed high sensitivity and specificity in measuring depressive episodes^39^.

### Covariates

Potential confounders were chosen based on review of the literature and restricted to variables available at all phases used in the analyses. All estimates were initially adjusted for age, ethnicity (White/ South Asian/ Black) and sex, with an interaction of sex and age where both sexes included. Socio-demographic variables consisted of marital status (married/cohabiting, single or divorced/widowed) and last employment grade level within the civil service, (high, intermediate, low). Health behaviours included smoking (never, former, current), alcohol intake (none: ≤1 unit/weeks, moderate, heavy: ≥14 units/week) self-reported physical activity (vigorous, moderate and non/mild)^40^ and duration of sleep (5 categories from ≤5 hours to ≥9 hours/day). Diet-related factors comprised energy intake, diet quality, fish, coffee and tea intake based on FFQ data. Energy intake was used to ascertain dietary misreporting. Misreporting was considered where the log ratio of energy intake to estimated energy expenditure was outside of 3 SD of the log mean. This definition was adopted by Mosdol *et al*. 2007 and based on basal metabolic rate equations of the Department of Health^41–43^. Since sugar intake from sweet food/beverages was strongly correlated with energy intake (r = 0.61, *P* < 0.001), energy intake was adjusted for with the partition method by using energy intake from other foods^44^. Diet quality was assessed using the Dietary Approaches to Stop Hypertension (DASH) diet score modified by excluding a measure for sweet drinks^45^. DASH diet score, coffee and tea intake were analysed as continuous variables, fish intake per day as quintiles and all dichotomized for descriptive analyses. Body mass index (BMI) (kg/m^2^) and central obesity (in women waist circumference ≥88 cm and in men ≥102 cm) were both measured by trained staff^46^. Physical health was defined as diabetes and cardiovascular disease (coronary heart disease and stroke, CVD) based on self-reports which were validated using the study clinical examination, Hospital Episode Statistics data, and by contacting general practitioners for confirmation when no other external source existed. Cancer was based on cancer registration data^29^. Finally, doctor diagnosis of depression was based on self-report at phases 1 to 4 and on self-reported antidepressant intake at all phases after phase 4.

### Statistical analysis

At each phase, participants were included if they had answered at least 8 of the FFQ sweet food and beverage items^47^ (less than 5% of eligible sample had one missing item and about 1% two or more), their ethnicity was known to be either White, Black or South Asian, and participants were not energy misreporters (see above). In addition, participants were also excluded from analyses if they had incomplete data on GHQ-, CES-D- or CIS-R caseness for outcome-specific analyses, respectively. Supplementary Fig. S1 shows how the included sample was reached (see Online).

**Figure 1:**
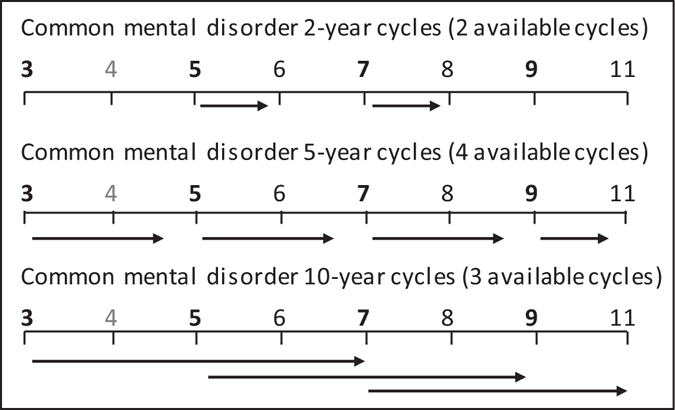
Modes of analysis using cycle approach for common mental disorder^a^. Numbers indicate study phases. Phases with food frequency data in bold; no data on common mental disorder available at Phase 4. ^a^Common mental disorder measured using the 30-item General Health Questionnaire.

Three binary outcomes were analysed, GHQ caseness, CES-D caseness and CIS-R caseness. Daily sweet food and beverage intake was modelled as sex-specific tertiles of sugar intake from sweet food/beverages based on the distribution at phase 3 (in men <39.5, ≥39.5 to <67.0 and ≥67.0 g/day; in women <30.0, ≥30.0 to <51.0 and ≥51.0 g/day). To describe the sample at phase 3, GHQ cases, non-cases and tertiles of sugar intake by covariate were compared. To examine the prospective association of sugar intake from sweet food and beverages, a random effects logistic regression model (REM) was performed using the STATA command *xtlogit* ^48^, with exposures at phases 3, 5, 7 and 9 for GHQ caseness, and at phases 7 and 9 for CES-D caseness. The applicability of the REM was tested by introducing study phase-interactions and likelihood ratio tests (LRT). The prospective effect of sugar intake from sweet food/beverages on incident and recurrent CMD and depression was examined using REMs in 2, 5 and 10-year cycles^49^. Figure 1 shows the included phases for analyses using GHQ caseness as the outcome. For example, the association between sugar intake and GHQ status 2 years later was conducted by combining the associations between sugar intake at Phase 5 and incident GHQ caseness at Phase 6, and between sugar intake at Phase 7 and incident GHQ caseness at Phase 8. For all depression outcomes, incidence was assumed if no CMD was apparent at each baseline, and recurrence if CMD was apparent at each baseline. For the analyses of depression, two 5-year cycles (to Phase 9 and 11) and three 10 year cycles (to Phases 7, 9 and 11) were used. For clinical depression, one 5-year cycle and one 10-year cycle were used.

To check for reverse causation, that depressive symptoms may affect subsequent sugar intake from sweet food/beverages, linear regression models of 5-year change and multinomial logistic regression for change groups were fitted for each cycle, from phases 3 to 5, 5 to 7 and 7 to 9, with CMD at phases 3, 5, 7 respectively, and for change from phase 7 to 9 with depression at phase 7. Normal distribution of change in sugar intake from sweet food/beverages was verified using a histogram. Change groups were created by subtracting tertiles of sugar intake at baseline (t) from sugar intake from sweet food/beverages at follow-up (t + 5 y) and coding −2/−1 as decrease, 0 as no change and +1/+2 as increase in sugar intake from sweet food/beverages.

All analyses were performed using Stata 14^50^. Interactions of CMD and depression with sex in the initial model (Model 0 per sex-specific tertile trend: adjusted for age and ethnicity) were tested using LRT since sex-differences have been reported in a prior study on the association of diet and depression in the Whitehall II cohort^51^. Further adjustments were grouped into four hierarchical models: baseline socio-demographic factors and health behaviours (Model 1), diet-related factors (Model 2), BMI and central obesity (Model 3), and physical health (Model 4). In sensitivity analyses, main analyses were repeated by: (a) excluding participants with unknown or reported doctor diagnosis of depression at each baseline (at phases 3/5/7/9: 166/156/193/209 individuals) and: (b) excluding participants with extreme values of sugar intake (>7 SD) at phases 3/5/7/9: 5/3/4/4 individuals.

## Results

Table 1 shows the prevalence of CMD and tertiles of reported sugar consumption from sweet food/beverages according to covariates at phase 3. CMD was more prevalent in women: under 50-years old, divorced/widowed, physically inactive, current smokers and those with fewer hours of sleep. Women with CMD were more likely to be in a lower grade level in civil service (*P* < 0.001; not depicted). Sugar consumption was associated with socio-demographic factors, health behaviours, physical health and diet-related factors (Table 1). Unexpectedly, participants in the highest tertile of sweet food/beverage intake had the highest prevalence of normal weight and lowest prevalence of overweight and obesity as well as the lowest prevalence of abdominal obesity in men (both *P* = 0.002; not depicted).

**Table 1:** Crude association of common mental disorder and sugar intake from sweet food/beverages with covariates at phase 3.

Covariates at phase 3	n	Common mental disorder cases^a^		Sugar intake from sweet food/beverages			
		%	*P*	Tertile 1	Tertile 2	Tertile3	*P*
				%	%	%	
Sex			<0.001				0.84
Men	5,603	20.7		69.0	69.7	69.1	
Women	2,484	25.7		31.0	30.3	30.9	
Age			<0.001				0.87
<50 years	4,264	25.3		53.1	52.5	52.6	
≥50 years	3,823	18.9		46.9	47.5	47.4	
Ethnic Group			0.03				
White	7,423	22.4		89.3	91.5	94.6	<0.001
South Asian	410	23.7		7.07	5.20	2.95	
Black	254	15.7		3.63	3.33	2.47	
Marital Status			<0.001				<0.001
Married/cohabiting	6,197	20.7		75.9	78.8	75.5	
Single	1,145	25.8		14.1	12.9	16.7	
Divorced/widowed	705	29.9		10.0	8.36	7.82	
Last grade level in Civil service			0.19				0.03
Highest	3,119	21.8		36.3	39.7	39.7	
Intermediate	3,639	23.1		46.0	44.2	44.8	
Lowest	1,329	20.8		17.7	16.1	15.5	
Smoking			0.006				<0.001
Never Smoker	3,540	21.0		42.7	47.6	49.4	
Ex-Smoker	2,957	22.4		41.9	38.3	36.5	
Current Smoker	1,108	25.5		15.4	14.2	14.2	
Physical activity			<0.001				0.002
Non/mild	3,027	25.4		40.3	36.6	35.4	
Vigorous	1,475	19.1		17.9	17.8	19.0	
Alcohol consumption			0.94				<0.001
None	1,916	22.4		20.8	22.3	28.0	
Moderate	4,208	22.1		48.3	53.8	54.1	
Heavy	1,955	22.4		30.9	23.9	17.9	
Sleep duration			<0.001				0.33
less than 7 h/day	2,055	27.3		26.4	24.6	25.4	
≥7 h/day	6,022	20.5		73.6	75.4	74.6	
Energy intake from other diet			0.03				<0.001
<median (1339 kcal)	4,042	21.2		61.8	52.6	35.7	
>median (2107 kcal)	4,045	23.3		38.2	47.4	64.3	
Modified DASH diet score			0.71				<0.001
<median (17.7)	4,522	22.1		50.6	55.6	61.7	
>median (24.8)	3,559	22.4		49.4	44.4	38.3	
Fish intake (			0.38				0.02
<median (1 portion/week)	4,597	21.9		56.6	58.9	55.1	
>median (4 portions/week)	3,489	22.7		43.4	41.1	44.9	
Coffee and tea			0.91				<0.001
≤1 cup of either/day	714	22.4		11.0	8.54	6.96	
>1 cup of either/day	7,373	22.2		89.0	91.5	93.0	
Body mass (BMI)			0.02				<0.001
Normal weight (<25 kg/m^2^)	4,052	22.7		48.2	52.0	57.7	
Overweight (25–29.9 kg/m^2^)	2,905	20.4		40.4	38.9	34.0	
Obese (≥30 kg/m2)	737	24.3		11.4	9.04	8.33	
Central obesity			0.59				0.21
No	6,938	21.9		90.4	91.1	91.8	
Yes (Waist ≥88 (F)/102 (M))	679	22.8		9.61	8.94	8.20	
Diabetes			0.90				<0001
No	7,861	22.2		96.1	97.4	98.1	
Yes	226	22.6		3.92	2.58	1.88	
CVD			0.03				0.007
No	7,817	22.1		95.8	96.7	97.3	
Yes	273	27.5		4.18	3.29	2.65	
Cancer			0.58				0.32
No	7.973	22.3		98.7	99.0	98.5	
Yes	105	20.0		1.33	1.05	1.51	

Abbreviations: SD = Standard Deviation, CVD = cardiovascular disease, DASH = Dietary Approaches to Stop Hypertension. *P* for difference or heterogeneity, derived from t-test, chi-square test, ANOVA or Kruskal-Wallis test. ^a^Common mental disorder measured using the 30-item General Health Questionnaire.

Incidence of CMD was around 9 to 15%, highest in the first cycle but did not differ greatly by cycle length. Depression and clinical depression incidence were approximately 8% and 2%, respectively. About 44% of participants who were CMD cases at baseline of each cycle remained recurrent CMD cases, 47% became recurrent depression cases and 58% recurrent clinical depression cases.

### Cross-sectional results

Cross-sectional analyses showed strong positive associations between sugar intake from sweet food/beverages and common mental disorder from the GHQ, as well as CES-D caseness, when adjusted for age, sex and ethnicity (Table 2). There was no evidence for any interaction with sex (*P* = 0.8 for GHQ and *P* = 0.7 for CES-D). The association with CMD was robust whereas for depression it was removed on adjustment for socio-demographic factors, health behaviours and diet-related factors (Table 2). Further adjustments for central obesity and physical health (not shown), exclusion of 709 person-observations (377 in CES-D analysis) with reported doctor diagnosis of depression and person-observations with extreme values of sugar intake at baseline did not change the results.

**Table 2:** Cross-sectional association of sugar intake from sweet food/beverages and prevalent common mental disorder and depression in men and women^a^.

	Prevalent common mental disorder^b^, OR (95% CI)			
	events/person observations	Model 0^c^	Model 1^d^	Model 2^e^
Sugar intake from sweet food/beverages				
Lowest Tertile	1540/8402	1.0 (reference)	1.0 (reference)	1.0 (reference)
Middle Tertile	1417/7439	1.08 (0.97, 1.20)	1.10 (0.99, 1.22)	1.07 (0.96, 1.19)
Highest Tertile	1435/6872	1.22 (1.09, 1.36)	1.25 (1.11, 1.41)	1.17 (1.04, 1.32)
Total	4392/22713			
*P* for trend		0.001	<0.001	0.011
	**Prevalent depression** ^**f**^ **, OR (95% CI)**			
	**events**/**person observations**	**Model 0** ^**g**^	**Model 1** ^**d**^	**Model 2** ^**e**^
Lowest Tertile	498/4025	1.0 (reference)	1.0 (reference)	1.0 (reference)
Middle Tertile	360/3186	1.04 (0.83, 1.30)	0.97 (0.77, 1.21)	0.90 (0.72, 1.14)
Highest Tertile	371/2684	1.36 (1.07, 1.73)	1.25 (0.98, 1.59)	1.08 (0.84, 1.39)
Total	1229/9895			
*P* for trend		0.016	0.098	0.643

Abbreviations: OR = Odds ratio, CI = Confidence interval, DASH = Dietary Approaches to Stop Hypertension. ^a^Cross-sectional association across phases 3, 5, 7, 9 for common mental disorder and 7, 9 for depression. ^b^Common mental disorder measured using the 30-item General Health Questionnaire.; ^c^CMD model 0 (4675 events/23954 person observations): adjusted for age*sex, ethnicity. ^d^Model 1: additionally adjusted for marital status, last grade level in civil service, smoking, alcohol intake, physical activity, sleep duration. ^e^Model 2: additionally adjusted for energy intake from other foods, modified DASH diet score, fish, coffee and tea intake. ^f^Depression measured using 20-item the Centre of Epidemiologic Studies Depression Scale. ^g^Depression model 0 (1313 events/10269 person observations): adjusted for age*sex, ethnicity.

### Prospective results

Prospective analyses regarding incident CMD were stratified by sex, since interactions with sex were observed in the 5 years later model (LR test for sex interaction: GHQ 2 years later, *P = *0.26: GHQ 5 years later, *P* = 0.05). In women, no associations were found for incident CMD with tertiles of sugar intake from sweet food/beverages (after 2 years, highest vs. lowest tertile OR: 0.98; 95% CI 0.72, 1.34; *P* for tertile trend = 0.90; after 5 years, highest vs. lowest tertile OR: 0.94; 95% CI 0.74, 1.19; *P* for tertile trend = 0.59). In men, after adjustment for age and ethnicity, sugar intake was associated with incident CMD 2 and 5 years later (Table 3). In further models, the association with 2-year incidence attenuated but the association with 5-year incidence remained (Table 3) and further adjustments for BMI, central obesity and physical health (not shown) resulted in an OR for highest vs. lowest tertile of: 1.23, 95% CI: 1.02, 1.48, *P* for trend = 0.03. Excluding participants who reported a doctor diagnosis of depression at each baseline strengthened the association (Model 4 for CMD after 5 years, Person observations = 10944; highest vs. lowest tertile OR; 1.25; 95% CI 1.03, 1.50; *P* for trend = 0.02, Supplementary Table S2) and exclusion of person observations with extremely high sugar intakes did not affect the results. In men and women, no association between sugar intake from sweet food/beverages and incident depression or clinical depression 5 years later was observed (Model 0, highest vs. lowest tertile OR, depression 0.92; 95% CI: 0.71, 1.18; *P* for tertile trend = 0.44; clinical depression: 0.95; 95% CI: 0.51, 1.75; *P* for trend = 0.84). The same exposure contrast was not associated with incident CMD or depression caseness after 10 years (Model 0, highest vs. lowest tertile OR: 1.10; 95% CI: 0.92, 1.31; *P* for tertile trend = 0.31; and 1.17; 95% CI: 0.91, 1.50, *P* for tertile trend = 0.25, respectively). However, sugar intake was positively associated with incident clinical depression after 10 years in men (Model 2 Person observations = 2572, cases = 35; *P* for tertile trend = 0.67), but negatively in women (Person observations = 848, cases = 28; *P* for tertile trend* = *0.02).

**Table 3:** Prospective association of sugar intake from sweet food/beverages and incident common mental disorder after 2 and 5 years in men^a^.

	Incident common mental disorder^b^ after 2 years, OR (95% CI)			
	events/person observations	Model 0^c^	Model 1^d^	Model 2^e^
Sugar intake from sweet food/beverages				
Lowest Tertile	220/2090	1.0 (reference)	1.0 (reference)	1.0 (reference)
Middle Tertile	205/1836	1.08 (0.85, 1.38)	1.08 (0.85, 1.38)	1.04 (0.81, 1.33)
Highest Tertile	202/1615	1.31 (1.02, 1.68)	1.30 (1.01, 1.67)	1.18 (0.90, 1.55)
Total	627/5541			
*P* for trend		0.039	0.047	0.233
	**Incident common mental disorder** ^**b**^ **after 5 years, OR (95% CI)**			
	**events**/**person observations**	**Model 0** ^**f**^	**Model 1** ^**d**^	**Model 2** ^**e**^
Lowest Tertile	477/4451	1.0 (reference)	1.0 (reference)	1.0 (reference)
Middle Tertile	446/3958	1.05 (0.90, 1.23)	1.07 (0.91, 1.25)	1.04 (0.88, 1.22)
Highest Tertile	463/3532	1.26 (1.07, 1.48)	1.28 (1.08, 1.51)	1.20 (1.01, 1.43)
Total	1386/11941			
*P* for trend		0.006	0.005	0.047

Abbreviations: OR = Odds ratio, CI = Confidence interval, DASH = Dietary Approaches to Stop Hypertension.

^a^Prospective association across phases 3, 5 for 2-year and 3, 5, 7, 9 for 5-year incident common mental disorder. ^b^Common mental disorder measured using the 30-item General Health Questionnaire.

^c^2-year model 0 (655 events/5767 person observations): adjusted for age and ethnicity. ^d^Model 1: additionally adjusted for marital status, last grade level in civil service, smoking, alcohol intake, physical activity, sleep duration. ^e^Model 2: additionally adjusted for energy intake from other foods, modified DASH diet score, fish, coffee and tea intake. ^f^5-year model 0 (1448 events/12445 person observations): adjusted for age and ethnicity.

Prospective analyses regarding the associations of sugar intake from sweet food/beverages and recurrent mood disorders showed no evidence for sex interaction for CMD, CES-D depression or clinical depression 5 years later. Sugar intake from sweet food/beverages was positively associated with recurrent depression after 5 years (Model 0, highest vs. lowest tertile OR: 1.81; 95% CI: 1.23, 2.66; *P* for tertile trend = 0.003, Table 4). The association was attenuated when adjusted for other diet-related factors. Moreover, there was some evidence that sugar intake from sweet food/beverages was associated with recurrent clinical depression in both sexes combined (highest vs. lowest tertile OR: 1.66; 95% CI: 0.96, 2.87 and *P* for tertile trend = 0.07) when adjusted for age, sex and ethnicity (Supplementary Table S3). This association attenuated when further factors were introduced to the model. No statistically significant association was found for sugar intake from sweet food/beverages and recurrent GHQ caseness after 2 and 5 years (Model 0 for CMD after 2 years, highest vs. lowest tertile OR: 1.05; 95% CI 0.76, 1.45; *P* for tertile trend = 0.83; for CMD after 5 years: 1.16; 95% CI 0.93, 1.46; *P* for tertile trend = 0.20).

**Table 4:** Prospective association of sugar intake from sweet food/beverages and recurrent depression after 5 years^a^.

	Recurrent depression after 5 years, OR (95% CI)^b^			
	events/person observations	Model 0^c^	Model 1^d^	Model 2^e^
Sugar intake from sweet food/beverages				
Lowest Tertile	258/848	1.0 (reference)	1.0 (reference)	1.0 (reference)
Middle Tertile	220/737	1.19 (0.83, 1.73)	1.10 (0.76, 1.60)	1.05 (0.72, 1.53)
Highest Tertile	263/719	1.81 (1.23, 2.66)	1.60 (1.08, 2.37)	1.47 (0.98, 2.22)
Total	741/2304			
*P* for trend		0.003	0.017	0.071

Abbreviations: OR = Odds ratio, CI = Confidence interval, DASH = Dietary Approaches to Stop Hypertension, CVD = cardiovascular disease. ^a^Prospective association across phases 7, 9 for recurrent depression. ^b^Depression measured using 20-item the Centre of Epidemiologic Studies Depression Scale.

^c^Model 0 (792 events/2435 person observations): adjusted for age*sex, ethnicity. ^d^Model 1: additionally adjusted for marital status, last grade level in civil service, smoking, alcohol intake, physical activity, sleep duration. ^e^Model 2: additionally adjusted for energy intake from other foods, modified DASH diet score, fish, coffee and tea intake.

Analyses of recurrent CMD, depression and recurrent clinical depression after 10 years showed no associations with sugar intake from sweet food/beverages.

Sensitivity analyses excluding extreme values of sugar intake and excluding person-observations with self-reported doctor diagnosis at baseline attenuated the association of sugar intake from sweet food/beverages and recurrent depression slightly (before *P* for tertile trend 0.003 after 0.022 and 0.010, respectively). Similarly, associations with clinical depression weakened when participants with depression diagnosis at baseline were excluded (Model 0, Person observations = 573; cases = 78; *P* for tertile trend = 0.17).

### Analysis of reverse causation

Sugar intake from sweet food/beverages decreased by 2.00 (SD 28.8; 95% CI 1.20, 2.79) grams per day from phase 3 to 5, by 3.44 (SD 28.0; 95% CI 2.59, 4.30) grams from phase 5 to 7 and by 1.57 (SD 26.0; 95% CI 0.91, 2.33) grams from phase 7 to 9, and was normally distributed. Mean 5-year change was approximately 31 g sugar from sweet food/beverages per day in the decrease group, −0.7 g in the stable intake group and 29 g in the increase group. Neither CMD, nor depression predicted 5-year changes in sugar intake (Table 5).

**Table 5:** Association of common mental disorder and depression with subsequent 5-year change in sugar intake from sweet food/beverages.

	5-year change in sugar intake			
	No. of events	Participants	OR (95% CI) *β*-*Coefficient* ^a^ *(95% CI)*	*P*
Common mental disorder^b^				
At phase 3 – Sugar intake change: phase 3 to 5				
Reduction	268	1201	1.0 (reference)	
No change	584	2860	0.91 (0.77, 1.08)	0.27
Increase	198	961	0.91 (0.74, 1.12)	0.39
Continuous change in grams per day	1050	5022	*0.08* (−*1.89, 2.05*)	0.94
At phase 5 – Sugar intake change: phase 5 to 7				
Reduction	210	1025	1.0 (reference)	
No change	464	2410	0.93 (0.77, 1.11)	0.41
Increase	176	734	1.21 (0.96, 1.53)	0.10
Continuous change in grams per day	850	4169	*1.18* (−*0.97, 3.33*)	0.28
At phase 7 – Sugar intake change: phase 7 to 9				
Reduction	200	744	1.0 (reference)	
No change	505	2206	0.87 (0.73, 1.05)	0.15
I ncrease	150	692	0.84 (0.66, 1.06)	0.14
Continuous change in grams per day	855	4497	−*1.14* (−*3.09, 0.82*)	0.26
Depression^c^				
At phase 7 – Sugar intake change: phase 7 to 9				
Reduction	116	764	1.0 (reference)	
No change	340	2233	1.05 (0.83, 1.32)	0.70
Increase	97	688	0.98 (0.73, 1.32)	0.90
Continuous change in grams per day	533	4238	*1.01* (−*1.35, 3.36*)	0.40

Abbreviations: No. = number, CI = confidence interval.

^a^Change in sugar intake in cases compared with non-cases, adjusted for age, sex and ethnicity. ^b^Common mental disorder measured using the 30-item General Health Questionnaire. ^c^Depression measured using 20-item the Centre of Epidemiologic Studies Depression Scale.

## Discussion

The present long-term prospective study is the first to investigate the association of sugar consumption from sweet food/beverages with prevalent, incident and recurrent mood disorders, while also examining the effect these disorders might have on subsequent habitual sugar intake. We found an adverse effect of higher sugar intake on mental health cross-sectionally and 5 years later in a study based on 23,245 repeated measures in men and women aged between 39 and 83. Further, we found an increased likelihood for incident CMD in men and some evidence of recurrent depression in both sexes with higher intakes of sugar from sweet food/beverages. These associations with incident CMD could not be explained by socio-demo graphic factors, other diet-related factors, adiposity and other diseases although the association with recurrent depression was explained by other diet-related factors.

In our study we were able to exclude potential ‘reverse causation’ as the reason for the observed link between high sugar intake and low mood. Over years and decades, it could be that those susceptible to depression tend to increase their sugar intake. This group may tend to report higher consumption at study baseline even in the absence of depression at the time of the questionnaire, while having an increased risk of future depression compared to other participants^14, 15^. However, there was no support for this alternative hypothesis, since the observed associations in our analysis were not the result of secondary changes in consumption of sugary food and drinks. Our study findings are consistent with the hypothesis that high sugar intake plays a causal role in the risks of both incident and recurrent depression and CMD.

Higher sugar intake from sweet food/beverages was associated with increased likelihood of incident CMD after 5 years in men. The association in men was in line with results from previous prospective studies in American and Spanish cohorts^9–11^. There are several potential explanations for the observed sex differences. First, the associations in men for incident CMD show a stronger effect with a bigger sample (comparing analysis of 2-year CMD with 5-year CMD), suggesting the lower number of female participants in our sample could have impaired the power of the analysis. Second, the results might reflect differences in pathways of depression by sex and type of depressive symptomatology^52–54^. Third, differences could be due to limitations of the study or to chance.

As described in the Introduction there are four potential mechanisms for an association of habitual sugar intake and subsequent depression risk. Sugar intake could increase depression risk over its potential influence on BDNF levels^16^ and inflammation^20^ which are both discussed as potential biological explanations for depression^17, 21^. Furthermore postprandial hypoglycaemia^22^ and addiction-like effects of sugar influencing neurotransmitters^23–25^ could link sugar intake with low mood. The pathway of postprandial hypoglycaemia is also relevant in the context of Glycaemic index, which has been shown to be associated with depression prevalence and incidence^55, 56^. However, it is a complex issue to tease apart the effects of a single nutrient in epidemiological studies since foods represent a mix of macro- and micronutrients. In this study associations were attenuated when adjusted for diet-related factors providing evidence of confounding and suggesting that the effect of sugar intake from sweet food/beverages could be partly explained by other components of the diet. Also, given that we analysed sugar intake from aggregated sweet foods and beverages we cannot rule out that certain types of foods and their particular components such as saturated fat content may have affected our findings. In our analysis, the association of sugar intake and recurrent depression was attenuated by measures of body fatness in participants without doctor diagnosis of depression at baseline supporting the hypothesis of an indirect effect mediated by adiposity^26–28^ driving the association of sugar intake and recurrent depression.

Meanwhile, there are several sources of possible error. Our study was based on an occupational cohort but sugar intakes from sweet food/beverages were close to those reported previously in a representative cohort in the UK (approximately 40 grams), and Batty *et al*. showed that effects found in Whitehall II were comparable to those observed in population-representative cohorts^3, 57^. A major limitation was the use of FFQ to derive diet data. FFQ data is subject to misreporting and underreporting, which have been found to differ by food group, depressive mood and BMI^58–60^. As reported previously in Whitehall, we showed a clear trend of lower sugar intake with higher BMI in men^60^. Nutrient content was based on food composition tables from 1991 and has to be considered as a source of error, since food composition especially of highly processed food is likely to change over the course of 18 years. In this long-term follow-up study, sugar intake from sweet food/beverages, which are consistently high in sugar content, has been used as the exposure measure. Compared to a measure of intake that includes processed foods^61^, this method may involve less information bias. Furthermore, this FFQ is meant to reflect habitual diet over the course of a year and therefore might not pick up short-term diet changes or occasional binge eating^30^. Although we adjusted for a number of potential confounders, we cannot rule out residual confounding through unknown or unmeasured factors. Finally, not all depression measures were obtained in all phases and selective dropout due to depressive symptoms might have influenced case numbers^62^.

In conclusion, our study provides evidence that sugar intake from sweet food/beverages increases the chance of incident mood disorders in men and limited evidence regarding recurrent mood disorders in both sexes. With a high prevalence of mood disorders, and sugar intake commonly two to three times the level recommended, our findings indicate that policies promoting the reduction of sugar intake could additionally support primary and secondary prevention of depression. To elucidate the association further, especially regarding observed sex differences our study should be replicated in representative prospective cohorts.

## Electronic supplementary material

Supplementary Information
