# Supplementary material for: Sugar intake from sweet food and beverages, common mental disorder and depression: prospective findings from the Whitehall II study
Source: Sci Rep. 2017 Jul 27;7:6287. doi: 10.1038/s41598-017-05649-7 (PMC5532289; doi:10.1038/s41598-017-05649-7)
Supplement: Supplementary file 1 — Supplementary Information [file 41598_2017_5649_MOESM1_ESM.pdf]

Sugar intake from sweet food and beverages, common mental disorder and depression: prospective findings from the Whitehall II study

Anika Knüppel[1*]

Martin J. Shipley[1]

Clare H. Llewellyn[1]

Eric J. Brunner[1]

[1]Affiliations: Department of Epidemiology and Public Health, University College London, London, WC1E 6BT, UK

*corresponding author: anika.knuppel.14@ucl.ac.uk

Participants at phases 3 / 5 / 6 / 7 / 8 / 9 / 11,
n=8815 / 7870 / 7355 / 6967 / 7173 / 6761 / 6308

Excluded: ethnicity unknown or not White / South Asian / Black,
n=104 / 66 / 66 / 65 / 73 / 63 / 52

Participants at Phases 3 / 5 / 7 / 9, n=8711 / 7804 / 6902 / 6698

Excluded:
- Missing values energy intake, n = 456 / 2410 / 1253 / 1324
- Missing information to assess energy misreporting, n = 86 / 107 / 59 / 47
- Energy (log EI/EE >3SD, <3SD), n = 66 / 28 / 36 / 30
- Less than half of sweet food/beverage items answered, n = 5 / 21 / 18 / 11

Participants with sufficient data on sugar intake from sweet foods/beverages:
Phases 3 / 5 / 7 / 9, n = 8098 / 5227 / 5534 / 5282

Excluded:
- Missing GHQ caseness, n = 11 / 81 / 50 / 27
- Missing CES-D caseness at phases 7 / 9, n = 341 / 178

Observations eligible for cross-sectional analysis:
For common mental disorder: Phases 3 / 5 / 7 / 9, n = 8087 / 5131 / 5483 / 5253
For depression: Phases 7 / 9 , n = 5175 / 5094

Observations eligible for incidence and recurrence analyses (based on common mental disorder at baseline):

	Follow-up period	Outcome phases	Incidence	Recurrence
GHQ	2 year	6 / 8	n = 3640 / 4178	n = 917 / 999
GHQ	5 year	5 / 7 / 9 / 11	n = 5116 / 3664 / 4069 / 4161	n = 1447 / 956 / 965 / 662
GHQ	10 year	7 / 9 / 11	n = 4850 / 3526 / 3806	n = 1397 / 925 / 917
CES-	5 year	7 / 9 / 11	n = 3366 / 3951 / 3996	n = 868 / 937 / 630
CES-	10 year	7 / 9 / 11	n = 4368 / 3416 / 3661	n = 1252 / 901 / 877
CIS-R	5 year	11	n = 3929	n = 631
CIS-R	10 year	11	n = 3514	n = 859

Figure S1 Inclusion of person observations by phase
Abbreviations: GHQ = General Health Questionnaire, CES-D = Centre of Epidemiologic Studies Depression Scale, CIS-R = revised Clinical Interview Schedule.

Sweet food	Beverages
sweet biscuits	fizzy soft drinks
buns or pastries	fruit squash or cordial
Cakes	fruit juice
chocolates or chocolate bars	malted milk drinks, such as Horlicks
fruit pies, tarts or crumbles	cocoa or hot chocolate
ice cream	
jam, marmalade or honey	
milk puddings, sponge puddings	
added sugar	
sweets, toffees or mints	

Table S1 Sources of sugar intake from sweet food/ beverages

	Incident common mental disorder[b] after 2 years, OR (95% CI)			
	events / person observations	Model 0[c]	Model 1[d]	Model 2[e]
Sugar intake from sweet food/beverages				
Lowest Tertile	214 / 2059	1.0 (reference)	1.0 (reference)	1.0 (reference)
MiddleTertile	194 / 1799	1.05 (0.82 ,1.34)	1.05 (0.82 ,1.34)	1.00 (0.78 ,1.29)
Highest Tertile	196 / 1581	1.33 (1.03 ,1.71)	1.31 (1.01 ,1.70)	1.19 (0.90 ,1.56)
Total	604 / 5439			
P for trend		0.032	0.045	0.234
	Incident common mental disorder[b] after 5 years, OR (95% CI)			
	events / person observations	Model 0[f]	Model 1[d]	Model 2[e]
Sugar intake from sweet food/beverages				
Lowest Tertile	463 / 4391	1.0 (reference)	1.0 (reference)	1.0 (reference)
MiddleTertile	434 / 3886	1.06 (0.90 ,1.24)	1.08 (0.92 ,1.27)	1.05 (0.89 ,1.24)
Highest Tertile	456 / 3467	1.30 (1.10 ,1.54)	1.32 (1.11 ,1.56)	1.24 (1.04 ,1.48)
Total	1353 / 11744			
P for trend		0.002	0.002	0.019

Table S2 Prospective association of sugar intake from sweet food/beverages and incident common mental disorder after 2 and 5 years in men excluding participants with self-reported doctor diagnosed depression at each baseline[a]

Abbreviations: OR = Odds ratio, CI = Confidence interval, DASH = Dietary Approaches to Stop Hypertension.
[a]Prospective association across phases 3, 5 for 2-year and 3, 5, 7, 9 for 5-year incident common mental disorder.
[b]Common mental disorder measured using the 30-item General Health Questionnaire.
[c]2-year model 0 (631events / 5661 person observations): adjusted for age, ethnicity.
[d]Model 1: additionally adjusted for marital status, last grade level in civil service, smoking, alcohol intake, physical activity, sleep duration.
[e]Model 2: additionally adjusted for energy intake from other foods, modified DASH diet score, fish, coffee and tea intake.
[f]5-year model 0 (1413 events / 12238 person observations): adjusted for age, ethnicity.

	Recurrent clinical depression after 5 years[b], OR (95% CI)		
		Model 0[c]	Model 1[d]	Model 2[e]
Sugar intake from sweet food/beverages				
Lowest Tertile	27 / 236	1.0 (reference)	1.0 (reference)	1.0 (reference)
MiddleTertile	26 / 182	1.37 (0.79, 2.40)	1.23 (0.68, 2.24)	1.11 (0.60, 2.05)
Highest Tertile	29 / 172	1.65 (0.96, 2.87)	1.58 (0.87, 2.86)	1.24 (0.67, 2.31)
Total	82 / 590			
P for trend		0.07	0.13	0.50

Table S3 Prospective association of sugar intake from sweet food/beverages and recurrent clinical depression after 5 years[a]

Abbreviations: OR = Odds ratio, CI = Confidence interval, DASH = Dietary Approaches to Stop Hypertension.
[a]Prospective association across phase 9 to 11.
[b]Clinical depression measured using the revised Clinical Interview Schedule.
[c]Clinical depression model 0 (92 events / 631 participants): adjusted for age*sex, ethnicity.
[d]Model 1: additionally adjusted for marital status, last grade level in civil service, smoking, alcohol intake, physical activity, sleep duration.
[e]Model 2: additionally adjusted for energy intake from other foods, modified DASH diet score, fish, coffee and tea intake.
